# Maternal Obesity in Early Pregnancy and Risk of Adverse Outcomes

**DOI:** 10.1371/journal.pone.0080410

**Published:** 2013-11-20

**Authors:** Inmaculada Bautista-Castaño, Patricia Henriquez-Sanchez, Nestor Alemán-Perez, Jose J. Garcia-Salvador, Alicia Gonzalez-Quesada, Jose A. García-Hernández, Luis Serra-Majem

**Affiliations:** 1 Department of Clinical Sciences, University of Las Palmas de Gran Canaria, Las Palmas de Gran Canaria, Spain; 2 Gynecology and Obstetrics Department, Maternal & Child University Hospital of Gran Canaria, Las Palmas de Gran Canaria, Spain; Paris Institute of Technology for Life, Food and Environmental Sciences, France

## Abstract

**Objectives:**

To assess the role of the health consequences of maternal overweight and obesity at the start of pregnancy on gestational pathologies, delivery and newborn characteristics.

**Methods:**

A cohort of pregnant women (n** = **6.558) having delivered at the Maternal & Child University Hospital of Gran Canaria (HUMIGC) in 2008 has been studied. Outcomes were compared using multivariate analyses controlling for confounding variables.

**Results:**

Compared to normoweight, overweight and obese women have greater risks of gestational diabetes mellitus (RR = 2.13 (95% CI: 1.52–2.98) and (RR = 2.85 (95% CI: 2.01–4.04), gestational hypertension (RR = 2.01 (95% CI: 1.27–3.19) and (RR = 4.79 (95% CI: 3.13–7.32) and preeclampsia (RR = 3.16 (95% CI: 1.12–8.91) and (RR = 8.80 (95% CI: 3.46–22.40). Obese women have also more frequently oligodramnios (RR = 2.02 (95% CI: 1.25–3.27), polyhydramnios. (RR = 1.76 (95% CI: 1.03–2.99), tearing (RR = 1.24 (95% CI: 1.05–1.46) and a lower risk of induced deliveries (RR = 0.83 (95% CI: 0.72–0.95). Both groups have more frequently caesarean section (RR = 1.36 (95% CI: 1.14–1.63) and (RR = 1.84 (95% CI: 1.53–2.22) and manual placenta extraction (RR = 1.65 (95% CI: 1.28–2.11) and (RR = 1.77 (95% CI: 1.35–2.33). Newborns from overweight and obese women have higher weight (p<0.001) and a greater risk of being macrosomic (RR = 2.00 (95% CI: 1.56–2.56) and (RR = 2.74 (95% CI: 2.12–3.54). Finally, neonates from obese mother have a higher risk of being admitted to special care units (RR = 1.34 (95% CI: 1.01–1.77). Apgar 1 min was significantly higher in newborns from normoweight mothers: 8.65 (95% CI: 8.62–8.69) than from overweight: 8.56 (95% CI: 8.50–8.61) or obese mothers: 8.48 (95% CI: 8.41–8.54).

**Conclusion:**

Obesity and overweight status at the beginning of pregnancy increase the adverse outcomes of the pregnancy. It is important to promote the normalization of bodyweight in those women who intend to get pregnant and to provide appropriate advice to the obese women of the risks of obesity at the start of the pregnancy.

## Introduction

The increase in the prevalence of obesity and overweight is affecting women of child-bearing potential (WOCBP) and is an increasing problem of public health. The mean body mass index (BMI) has increased in all the age categories, and women begin pregnancy with increased weight. A review published by Guelinckx et al [1] refers to a prevalence of obesity in pregnant women varying between 1.8% and 25.3%, depending on the country. A previously published study found a pregnant women overweight prevalence of 25% and an obesity prevalence of 17.1% in Gran Canaria [2].

Different studies have shown that compared with normal-weight women, obese women have a higher prevalence of infertility, recurrent miscarriage, congenital malformations and intrauterine fetal death [3], [4].

The excess weight in pregnancy is considered a high risk state because it is associated with many adverse complications obstetric and perinatal outcomes such as gestational diabetes, hypertension, preeclampsia, thromboembolism, macrosomia, higher incidence of caesarean deliveries and perinatal mortality [5]–[9]. Otherwhise, children with history of high birth-weight also have an elevated risk of developing obesity and type 2 diabetes later in life [10].

Another of the problems in pregnancy associated with excess body-weight is that the women tend to retain some of the weight-gain with each pregnancy [11]. According to the results of a meta-analysis published by Nehring et al [12] it has been found that, compared with women with gestational weight gain within the recommendations, those with a gestational weight gain above the recommendations retained an additional 3.06 kg after 3 years and 4.72 kg on average after 15 years postpartum.

As a consequence, women who start pregnancy are a target group to prevent excessive weight gain during pregnancy.

To our knowledge no studies have been published about the health consequences of maternal overweight and obesity at the start of pregnancy on gestational pathologies, delivery and newborn characteristics in the Canary Island, a Spanish community with a high obesity prevalence at the start of pregnancy [2] and therefore the objective of the present study was to investigate this relationship in women who delivered at the HUMIGC in 2008.

## Methods

### Ethics Statements

The present study was conducted according to the guidelines laid down in the Declaration of Helsinki. The protocol was approved by the Human Research Ethical Committee of the Maternal & Child University Hospital of Gran Canaria (HUMIGC). Participants provided a verbal consent to their Doctors during the physical examination. This consent was registered in the medical record of each patient. The study was submitted to the Ethics Committee of the Maternal & Child Hospital of Gran Canaria, as already stated. The mentioned Committee did not object the protocol since "no experimental procedures" and "no analytical samples" were included. The Ethical Committee knew that some of the participants were minors, and did not object to the approval of the consent procedure.

### Methods

A population-based retrospective cohort study of all the pregnant women having delivered at the HUMIGC in 2008, summing up 6,887 women, has been performed. This number represents approximately 90% of all the 2008 births (n = 7,729) at Gran Canaria [2].

194 participants without data or with incorrect data regarding weight, height and/or age and 135 participants with a multiple pregnancy have been excluded. Finally, 6,558 participants were included in this study.

Data on maternal characteristics and on pregnancy, birth and post-partum complications were retrieved from the clinical registries made at the Gynaecologic and Obstetrics HUMIGC Service.

The main predictor variable was maternal BMI in early pregnancy. BMI was categorized in the following groups, according to the Guidelines of American Clinics for the identification, evaluation and treatment of obesity and overweight in adults [13]: normoweight (BMI 18.5–24.9 kg/m^2^), overweight (BMI 25–29.9 kg/m^2^) and obese (BMI ≥30 kg/m^2^) (12). Underweight women (BMI <18.5 kg/m^2^) were excluded from multivariate analyses.

For further analyses, information about age, socio-cultural status (low: primary school studies or no formal education; middle: secondary school education; high: university education or equivalent), smoking status, pre-gestational pathologies (diabetes, hypertension, asthma) and previous gestational antecedents (infertility, premature delivery, abortion, perinatal death and congenital anomalies) has been collected.

Outcomes assessed were gestational pathologies, delivery and newborn characteristics.

Gestational pathologies included were diabetes, hypertension, preeclampsia, oligohydramnios (amniotic fluid index ≤ 5 cm), polyhydramnios (amniotic fluid index > 24 cm), threat of pre-term delivery (labor uterine contractions starting between 28^th^ and 37^th^ week), premature membrane rupture (PMR, spontaneous rupture of the fetal membranes before week 37 without labor work), placenta praevia and repeated urinary tract infections.

Delivery characteristics were dilation time**,** gestational age, type of delivery (caesarean, forceps, vaginal eutocic), delivery induction, placental extraction (Credé maneuver or manual), episiotomy, and vaginal tearing (ranked in four degrees (Grade 1: First degree injury to perineal skin only; Grade 2: injury to perineum involving perineal muscles but not involving the anal sphincter; Grade 3: injury to perineum involving the anal sphincter complex and Grade 4: injury to perineum involving the anal sphincter complex and anal epithelium).

Newborn characteristics were weight, macrosomia (weight >4000 g), Apgar 1, Apgar 5 pH, transferred (incubator, transition, neonate unit or special-care unit) and death (ante-partum or neonatal).

Concerning statistical analysis, for descriptive purposes, means, standard deviations, and proportions of characteristics at baseline across maternal categories of BMI have been calculated. Moreover, the association between maternal categories of BMI and characteristics at baseline, has been analyzed through ANOVA tests for means contrasts and Chi-squared test for proportions contrasts.

Ordinal logistic regression analyses calculating odds ratios (OR) and their 95% confidence intervals (95% CI) were fit to assess the association between gestational morbidity, partum and newborn delivery related (included abnormal Apgar score (<7)) and maternal categories of BMI, Normoweight women were used as the reference group. Potential confounders included as covariates in the model were maternal age (years, continuous), smoking habit (yes/no), socio-economic level (low, middle and high) and parity (yes/no).

Finally, generalized linear models were used to assess the association between BMI categories and neonatal weight, Apgar 1 min, Apgar 5 min, pH and dilation length adjusting for maternal age, socio-economic level, smoking and parity. Statistical differences in multivariate adjusted mean scores according to the different categories of BMI were determined by ANCOVA.

All P values presented are two-tailed and statistical significance was defined *a priori* at P<0.05. Data analyses were performed using SPSS 19.0 (SPSS Inc, Chicago, IL, USA).

## Results

The 6,558 women in the final sample had a mean age of 29.8±6.0 years (range: 14–52 years). Maternal BMI at the start of pregnancy was 25.2±5.3 kg/m^2^ (range: 14– 61). The systolic blood pressure (SBP) was 122.1±16.1 mmHg (range: 72–210) and diastolic blood pressure (DBP) was 73.8±11.3 mmHg (range: 40–168).

Of the overall sample, 41.5% were primigravida and 13.2% were smokers. With respect to socio-economic status, the distributions of low, middle and high were 46.7%, 34.2% and 18.3%, respectively.

The main characteristics of participants according to categories of BMI are shown in **table 1**. Participants with higher BMI have a higher prevalence of pre-gestational diabetes, pre-gestational hypertension and asthma. The largest difference was related to the prevalence of pre-gestational hypertension (6.3% obese women vs 0.4% normal weight women). No significant differences were found in relation to the socio-cultural level, smoking habit, percentage of primiparous or history of infertility.

**Table 1:** Baseline maternal characteristics and pregestacional pathologies by maternal categories of body mass index.

	Underweight	Normal	Overweight	Obese	p-value
	273 (4.2%)	3.534 (53.9%)	1.635 (24.9%)	1.116 (17%)	
Age (years), means (SD)	26.6 (6.2)	29.6 (6.1)	30.3 (5.9)	30.5 (5.8)	<0.001^a^
TAS (mm Hg), means (SD)	116.5 (15.4)	119.5 (14.9)	124.7 (16.2)	128.3 (17.5)	<0.001^a^
TAD (mm Hg), means (SD)	70.9 (10.4)	72.2 (10.9)	70.9 (10.4)	77.3 (11.9)	<0.001^a^
Socio-cultural status %					
High	15.4	18.8	19.2	19.2	0.215^b^
Middle	39.9	33.4	35.6	34.3	
Low	44.7	47.9	45.2	46.4	
Smoking %	9.2	13.6	13.3	12.7	0.199^b^
Primigesta %	39.9	40.9	42.9	41.8	0.544^b^
Pre-gestational diabetes %	0.4	0.5	0.6	1.3	0.028^b^
Pre-gestational hypertension %	0	0.4	1.3	6.2	<0.001^b^
Asthma %	1.1	1.5	1.9	3	0.01^b^
Infertility %	0.4	0.7	0.9	1	0.053^b^

a p from Anova test.

b p from χ^2^-test.

The weight gain during pregnancy was 11.79±5.31 kg with the values ranging between -5 and +391kg. Under and normoweight women show the greater tendency towards ponderal increase, compared to those who were overweight and obese (p<0.001).

Of the variables studied, it was observed that overweight and obesity increased the risk of suffering gestational DM, gestational hypertension, and preeclampsia. The risk of oligohydramnios and polyhydramnios was associated only with pregnancy in obese women (**table 2**).

**Table 2:** Gestational morbidity by maternal categories of body mass index$.

		Normoweight		Overweight/Normoweight			Obese/Normoweight	
		n = 3.534		n = 1.635			n = 1.116	
				RR	RR		RR	RR
				unadjusted	adjusted$		unadjusted	adjusted$
	n (%)		n (%)	(95%CI)	(95%CI)	n (%)	(95%CI)	(95%CI)
**Diabetes**	70 (2.0)	1 (ref)	71 (4.4)	2.25 (1.61–3.15)*	2.135 (1.52–2.98)*	63 (5.7)	2,99 (2.17–4.22)*	2.85 (2.01–4.04)*
**Hypertension**	38 (1.1)	1 (ref)	36 (2.2)	2.01 (1.32–3.31)*	2.01 (1.27–3.19)*	52 (5.0)	5.73 (3.89–8.41)*	4.79 (3.13–7.32)*
**Preeclampsia**	6 (0.2)	1 (ref)	9 (0.6)	3.26 (1,16–9.20)^#^	3.16 (1.12–8.91)^#^	17 (1.5)	9,10 (3.58–23.13)*	8.80 (3.46–22.40)*
**Oligohydramnios**	44 (1,2)	1 (ref)	24 (1.5)	1.18 (0.72–1.95)	1.17 (0.71–1.93)	29 (2.6)	2.12 (1.32–3.40)^#^	2,02 (1.25–3.27)^#^
**Polyhydramnios**	38 (1.1)	1 (ref)	27 (1.7)	1.55 (0.94–2.54)	1.43 (0.86–2.34)	22 (2.0)	1,85 (1.09–3.14)^#^	1.76 (1.03–2.99)^#^
**Premature delivery or placental**	122 (3.5)	1 (ref)	39 (2.4)	0.68 (0.47–0.98)^#^	0.70 (0.49–1.0)	30 (2.7)	0,77 (0.52–1.16)	0.8 (0.53–1.20)

$ Adjusted for maternal age, socio-economic level, smoking and gravidity.

* p<0.001.

# p< 0.05.

Of the pathologies studied, it was observed that obese and overweight women had an increased risk of caesarean delivery (adjusted OR of 1.36; 95% CI: 1.14–1.63 for overweight and OR of 1.84; 95% CI: 1.53–2.22 for obese) as well as a lower probability of having vaginal and forceps delivery (**table 3**). Induced delivery was significantly lower in obese women and the risk of manual extraction/delivery was increased not only in obese women but also in overweight individuals. The risk of second degree tearing increased in the obese group.

**Table 3:** Partum and newborn delivery-related morbidity by maternal categories of body mass index.

		Normoweight		Overweight/Normoweight			Obese/Normoweight	
		n = 3.534		n = 1.635			n = 1.116	
				RR	RR		RR	RR
				unadjusted	adjusted$		unadjusted	adjusted$
	n (%)		n (%)	(95%CI)	(95%CI)	n (%)	(95%CI)	(95%CI)
**Intra-partum**								
**Eutocic-head**	2736(77,4)	1 (ref)	1220 (74.6)	0.86 (0.75–0.98)#	0.89 (0.77–1.02)	803 (72.0)	0.75 (0.64–0.87)*	0.78 (0.67–0.91)*
**Forceps**	365 (10.3)	1 (ref)	150 (9.2)	0.88 (0.72–1.07)	0.87 (0.71–1.06)	92 (8.2)	0.78 (0.61–0.99)#	0.77 (0.60–1.00)#
**Caesarean**	380 (10.8)	1 (ref)	239 (14.6)	1.42 (1.19–1.69)*	1.36 (1.14–1.63)*	209 (18.7)	1.91 (1.59–2.30)*	1.84 (1.53–2.22)*
**Induced delivery**	2178(61.6)	1 (ref)	964 (59.0)	0.89 (0.79–1.00)	0.89 (0.79–1.005)	633 (56.7)	0.82 (0.71–0.93)*	0.83 (0.72–0.95)*
**Manual placenta extraction**	155 (4.4)	1 (ref)	116 (7.1)	1.66 (1.30–2.13)*	1.65 (1.28–2.11)*	85 (7.6)	1.80 (1.37–2.36)*	1.77 (1.35–2.33)*
**Delivery canal**								
**Grade 2 tearing**	699 (17.9)	1 (ref)	326 (19.5)	1.09 (0.94–1.27)	1.10 (0.95–1.28)	235 (21.3)	1.24 (1.05–1.46)*	1.24 (1.05–1.46)#
**Episiotomy**	1025(29.0)	1 (ref)	400 (24.5)	0.79 (0.69–0.91)*	0.79 (0.69–0.90)*	217 (19.4)	0.59 (0.509–0.70)*	0.59 (0.50–0.70)*
**Macrosomia**	142 (4.4)	1 (ref)	131 (8.7)	2.07 (1.62–2.65)*	2.00 (1.56–2.56)*	115 (11.7)	2.83 (2.19–3.66)*	2.74 (2.12–3.54)*
**Transferred/observation**	216 (5.6)	1 (ref)	102 (6.2)	1.12 (0.87–1.42)	1.08 (0.84–1.39)	83 (7.7)	1.41 (1.08–1.83)*	1.34 (1.01–1.77)*

$ Adjusted for maternal age, socio-economic level, smoking and gravidity.

* p<0.001.

# p< 0.05.

Among the alterations studied, it was observed that being obese or overweight at the start of pregnancy increased the risk of having an overweight baby (macrosomia), and being obese a higher risk of being transferred to special care and observation units.

**Table 4** shows the estimated multivariate-adjusted means (and their 95% CI) for neonate weigh, Apgar 1 min, Apgar 5 min, pH and dilatation length according to categories of maternal baseline body mass index.

**Table 4:** Multivariate adjusted mean values (95% confidence interval)$ on newborn characteristics by maternal categories of body mass index.

	Normoweight	Overweight	Obese	p
	n = 3.534	n = 1.635	n = 1.116	(ANCOVA)
**Neonate weight**	3173.0 (3154.0–3192.0)	3261.0 (3233.0–3288.4)	3299.6 (3265.7–3333.6)	<0.001
**Apgar 1 min**	8.65 (8.62–8.69)	8.56 (8.50–8.61)	8.48 (8.41–8.54)	<0.001
**Apgar 5 min**	9.41 (9.38–9.44)	9.33 (9.29–9.37)	9.26 (9.21–9.31)	<0.001
**pH**	720.4 (720.1–720.7)	720.4 (720.0–720.9)	719.9 (719.4–720.4)	0.175
**Dilatation length**	4.81 (4.62–5.0)	5.67 (5.39–5.95)	5.76 (5.42–5.95)	<0.001

$ Adjusted for maternal age, socio-economic level, smoking and gravidity.

Newborn weight was directly related to maternal baseline body mass index (p<0.001). Adjusted dilation length was increased in overweight and obese in relation to normoweight women (p<0.001). On the contrary, Apgar scores at 1 and 5 minutes were inversely related.

Overweight and obese women are at increased risk of having children with abnormal Apgar score (<7) at the first minute than women with normal weight, RR = 1.327 (95% CI 1.043–1.689) and RR = 1.777 (95% CI 1.382–2.286), respectively. However, the association becomes non-significant at the fifth minute, RR = 1.208 (95% CI 0.628–2.323) and RR = 1.658 (95% CI 0.847–3.246).

## Discussion

As previously reported [2], 25.0% of the studied sample of pregnant women were overweight and 17.1% were obese, increasing both with age and not influenced with the educational level. Among the values available from other European countries, only the UK reported values greater [1], [14].

The present study indicated, in concordance with other studies [15]–[17], that the risks of DM and hypertension before and during pregnancy are increased in obese and overweight women. A meta-analysis exploring the association between gestational DM and BMI estimated that the risk of developing gestational DM is two and four times higher among overweight and obese women respectively compared with normal-weight pregnant women [18]. Insulin resistance plays an important role in these pathologies. In normoweight women, pregnancy is already associated with a progressive decrease in insulin sensitivity during the pregnancy [19], [20]. This metabolic adjustment appears to be magnified in obese women. Not only the peripheral but also hepatic insulin resistance is seen to be increased in glucose-tolerant pregnant women, when compared with slim or normoweight women; peripheral insulin sensitivity is 40% less in obese women [21].

With respect to the gestational and pre-gestational pathology, no statistically significant relationships between obesity or overweight and repeated urinary tract infections were observed. However other studies, have shower an increased incidence of urinary tract infection in obese pregnant women [22], [23].

The present study showed an increased risk of preeclampsia in obese women. A meta-analysis of maternal BMI and preeclampsia showed that the risk was doubled with every 5 to 7 unit increase in BMI [24]. Despite this increased risk for preeclampsia, no increased risk of preterm delivery (both conditions are usually related to each other), was found. It would be very interesting to investigate which obesity-related factors could be involved in the relationship between preecampsia and premature delivery.

A meta-analysis of 11 cohort studies in pregnant women showed that the risk of Caesarean delivery increased by 50% in overweight women and more than doubled in obese women compared with normoweight women [25]. We have also observed an increased risk of Caesarean delivery in women with excess ponderal status.

Different factors could influence this increased risk. For example, obesity is associated with pregnancy complications, including pre-eclampsia, diabetes and gestational hypertension, induced labor, and delivery of a macrocosmic infant.

In line with other authors [5], [26] we observed a higher risk of fetal delayed-term macrosomia in women with overweight and obesity. The importance of the delayed-term fetus macrosomia is that the subsequent risk of infant and childhood obesity is also increased, and which is associated with insulin resistance, diabetes and hypertension over the long-term development of the child [10].

We also studied other newborn characteristics like newborn weight. Apgar 1, Apgar 5, pH, transferred (incubator, transition, neonate unit or special-care unit) and death (ante-partum or neonatal). We observed that newborn weight was directly related to maternal baseline body mass index, albeit the obese women had significantly less weight-gain compared to the normoweight individuals. This could indicate that the maternal fat deposits would influence the increase in basal energy metabolism, and which would prevent the unhealthy acquisition of additional adipose tissue [27].

Similarly newborn of overweight and obese mother, have greater risk of being admitted to special care units, according to their significant lower Apgar scores, which in turn has significant health economic effects. However, increased risks of factors leading to perinatal morbidity such as neonatal trauma and admission to special care unit have been reported in only a few studies [23], [28]–[30].

Despite this, it was found that overweight and obese women were not at increased risk of stillbirth in line with others authors [5], although a recent study [4] has found that BMI categories of 30–34.9 and 35 or more represented a 40% and 60% increased risk of stillbirth, respectively, and the study of Yu in 2006 showed an increased perinatal mortality (1.4 per 1000 versus 5.7 per 1000 in the obese group) [31].

The present study showed a high risk of a wide range of important maternal and neonate pathologies in women who began pregnancy in an obese or overweight condition. The etiology of this increase in risk is still not well defined although, probably, a wide range of metabolic, inflammatory and vascular factors are implicated together with social characteristics, genetic factors, inappropriate dietary habits and lack of physical activity.

Other interesting questions are the adverse effects that the excess maternal weight in pregnancy on child health in the long time may have. It is well documented that many of the risks associated with obesity at the start of pregnancy can increase the susceptibility of the fetus to disease in later ages [32]. Maternal obesity appears to be associated with a higher risk of childhood obesity [33], [34] and a cardiovascular and metabolic risk profile in childhood or early adulthood [35]. A recent review showed that High maternal BMI at both early and late pregnancy also increased risk of schizophrenia in the offspring [36]. Future studies should take into account other factors that might potentially mediate this association.

In line with the thinking of other authors [5], [7], [37], we believe that pre-natal care in women with excess weight would need to be individualized and monitored by a multi disciplinary team to reduce/control the risk and to improve maternal and fetal outcomes. Intervention studies need to be conducted in obese WOCBP with the intent of modifying the risk and to improving the outcomes of their future pregnancies. It is important to promote the normalization of bodyweight in those women who intend to get pregnant and to provide appropriate advice to the obese women of the risks of obesity at the start of the pregnancy. Increased maternal and neonatal morbidity results in the increased utilization of resources at a significant cost to the community and is presenting a critical challenge to healthcare services. There is an urgent need to establish effective preventative strategies, both prior to pregnancy and during pregnancy, based on evidence from high quality randomized controlled trials.
