# Correction: Maternal Obesity in Early Pregnancy and Risk of Adverse Outcomes

**DOI:** 10.1371/annotation/f8605b0a-d01c-41aa-ac9b-b605d7903a28

**Published:** 2013-12-19

**Authors:** Inmaculada Bautista-Castaño, Patricia Henriquez-Sanchez, Nestor Alemán-Perez, Jose J. Garcia-Salvador, Alicia Gonzalez-Quesada, Jose A. García-Hernández, Luis Serra-Majem

There were errors in affiliations and missing affiliations for multiple authors. Please see the list of authors with the correct indications of affiliations, as well as the correct list of affiliations, below:

Inmaculada Bautista-Castaño1,2, Patricia Henriquez-Sanchez1,2, Nestor

Aleman-Perez1, Jose J. Garcia-Salvador1, Alicia Gonzalez-Quesada1,

Jose A. Garcıa-Hernandez3, Luis Serra-Majem1,2

1 Department of Clinical Sciences, University of Las Palmas de Gran

Canaria, Las Palmas de Gran Canaria, Spain, 2 Biomedical Research

Center Network on Obesity and Nutrition (CIBERobn) Physiopathology of

Obesity and Nutrition, Institute of Health Carlos III, Madrid, Spain 3

Gynecology and Obstetrics Department, Maternal & Child University

Hospital of Gran Canaria, Las Palmas de Gran Canaria, Spain 

